# *In vivo* mosquito repellency effect of citronella (*Cymbopogon nardus* (L.) Rendle) essential oil bath bomb formulation in dogs

**DOI:** 10.14202/vetworld.2024.1538-1544

**Published:** 2024-07-13

**Authors:** Suwit Uopasai, Ketmanee Senaphan, Glenn Neville Borlace, Eakachai Thongkham, Jareerat Aiemsaard

**Affiliations:** 1 Division of Anatomy, Faculty of Veterinary Medicine, Khon Kaen University, Khon Kaen 40002, Thailand; 2 Division of Physiology, Faculty of Veterinary Medicine, Khon Kaen University, Khon Kaen 40002, Thailand; 3 Department of Pharmaceutical Chemistry, Faculty of Pharmaceutical Sciences, Khon Kaen University, Khon Kaen 40002, Thailand; 4 Division of Pharmacology and Toxicology, Faculty of Veterinary Medicine, Khon Kaen University, Khon Kaen 40002, Thailand

**Keywords:** bath bomb formulation, *Culex quinquefasciatus*, *Cymbopogon nardus*, mosquito repellent, vector control

## Abstract

**Background and Aim:**

Mosquitoes carry numerous diseases of medical and veterinary significance. While citronella essential oil is safe as a mosquito repellent, extensive research does not document its ability to deter mosquitoes from animals. This study assessed the citronella essential oil bath bomb’s ability to repel *Culex quinquefasciatus* mosquitoes in dogs.

**Materials and Methods:**

Citronella essential oil’s chemical composition was analyzed using gas chromatography-mass spectrometry (GC-MS). Through freeze-thaw testing, a bath bomb formulation containing 6% w/w citronella essential oil was assessed for its physical and chemical stability. Thirty-two healthy client-owned mixed-breed dogs were employed to test the mosquito-repellency effects of citronella essential oil (treatment group) and olive oil (control group) bath bomb formulations. Bath bombs were tested for irritation effects on animal skin for 15-day post-application.

**Results:**

Thirty-six compounds were identified through GC-MS, with citronellal (23.38%), δ-cadinene (12.25%), and geraniol (9.09%) being the most prevalent constituents. The bath bomb maintained its original physical properties after undergoing six freeze-thawing cycles and retained over 90% of its citronella essential oil. About 100%, 69.28%, and 65.58% mosquito repellency were displayed by the citronella essential oil bath bomb at 3 h, 6 h, and 8 h, respectively. None of the test animals exhibited skin irritation during the study.

**Conclusion:**

The citronella bath bomb effectively repelled *C. quinquefasciatus* in dogs without irritating their skin. The formulation’s physical and chemical stability is demonstrated by the results of freeze-thaw stability testing. Further studies should be conducted to evaluate the repelling activity against other mosquito species.

## Introduction

Mosquitoes carry significant medical and veterinary importance as temporary ectoparasites. In Thailand, *Culex* spp., *Aedes aegypti*, *Aedes albopictus*, *Anopheles* spp., *Mansonia* spp., and *Armigeres subalbatus* are the prevalent mosquito species. *Culex quinquefasciatus* was the dominant mosquito species across fragmented forests, rice fields, and rural and suburban environments [1]. Mosquito bites can trigger allergic reactions and excessive scratching in animals with sensitive skin, leading to hair loss or wounds. Many mosquito species are also carriers of *Dirofilaria immitis*, or heartworm (dirofilariasis), which is an important disease in dogs and cats in Thailand, South-east Asia, and South Asia [2]. Dogs as young as 2 months can become infected with *D. immitis*. Animals suffering from severe dirofilariasis may experience heart and liver failure [3]. Mosquitoes transmit *Brugia pahangi*, causing inflammation and lymphatic vessel blockage, as documented in Thailand [4, 5]. In animals with severe symptoms, dirofilariasis can cause heart and liver failure [3].

Thailand classifies insect and pest control products as hazardous substances subjected to legal registration [6]. These common pesticides, fipronil, allethrin, and permethrin, can impact not only insects’ nervous systems but also harm mammals, beneficial insects, and aquatic animals. These chemicals, when used properly in tick and flea treatments, are safe for animals. However, there are reports of adverse reactions such as pruritus and erythema (13%–24%) and gastric dilation and azotemia (1%–2%) [7, 8].

Citronella essential oil (*Cymbopogon nardus* (L.) Rendle) is an alternative mosquito repellent safe for humans and animals when used in appropriate concentrations. Citronella essential oil has been shown to be effective as a mosquito repellent in humans against *A. aegypti*, *C. quinquefasciatus*, and *Anopheles stephensi* [9], and its efficacy has been shown to be dependent in part on the type of formulation developed by Solomon *et al*. [10].

However, there are limited studies on the effectiveness of citronella essential oil in repelling mosquitoes from animals. Therefore, we developed a bath bomb formulation containing citronella essential oil suitable for bathing pet animals and assessed its repellency against *C. quinquefasciatus* in dogs.

## Materials and Methods

### Ethical approval

This study was approved by the Institutional Animal Care and Use Committee of Khon Kaen University and was based on the Ethics of Animal Experimentation of the National Research Council of Thailand (number IACUC-KKU-126/66).

### Study period and location

This study was conducted from September 2023 to April 2024 at the Faculty of Veterinary Medicine, Khon Kaen University, Thailand.

### Citronella essential oil and gas chromatography-mass spectrometry (GC-MS) analysis

Citronella essential oil extracted from *C. nardus* (L.) Rendle by steam distillation was purchased from Thai China Flavors and Fragrances Industry Co., Ltd., Ayutthaya, Thailand, batch no. 23091138-4. Chemical constituent analysis was conducted using an Agilent 6890 N Gas chromatograph equipped with a 5973 Mass Selective Detector (Agilent Technologies, Inc., USA). The column was a DB-5MS capillary GC column (30 m by 0.25 mm, film thickness 0.25 μm) containing 5% phenyl- 95% dimethylpolysiloxane fused silica. Helium was used as the carrier gas (1 mL/min, constant flow). The injected volume was 2 μL, and the temperature was started at 70°C, then increased at a rate of 2°C/min to a maximum of 220°C for 10 min. The inlet and ion source temperatures were 230°C and 280°C, respectively. The mass spectrum of the chemical constituents of citronella essential oil was compared with those of mass spectral libraries (Wiley7 n.1, John Wiley & Sons, Inc., USA) [11].

### Bath bomb formulation

The bath bomb base contained sodium bicarbonate (45% w/w), citric acid (45% w/w), and olive oil (10% w/w). All components were obtained from Union Science Trading Co., Ltd., Khon Kaen, Thailand. In the citronella bath bomb formulation, olive oil (6% w/w) was replaced with citronella essential oil (6% w/w). All ingredients were mixed and compressed using a round mold (35–40 g each).

### Stability testing of the bath bomb formulation

The formulation’s physical properties and essential oil content were assessed before and after six freeze-thaw cycles of 24 h at −5°C followed by 24 h at 40°C [12]. The physical properties of the formulations that were measured were pH (8% w/v dilution, Lab 850 set pH meter, SI Analytics, Germany) and color, which were assessed by visual observation. The citronella essential oil content was determined using a microplate spectrophotometer (Epoch™ 2, BioTek Instruments, Inc., USA). The bath bomb formulation was extracted with hexane 3 times (Brightchem Sdn Bhd, Malaysia) and filtered through Whatman filter paper no. 1. The extract was diluted two-fold with hexane in 96-well microtiter plates. Absorbance at 286 nm was compared with a citronella essential oil calibration curve [13].

### Animals

The sample size was derived from the data of a previous study conducted by Fankhauser *et al*. [14]. Thirty-two healthy mixed-breed dogs aged 2–8 years took part in the study. Animals underwent a drug-free month before the test. Larval stage *C. quinquefasciatus* was obtained from the Department of Medical Sciences, Ministry of Public Health, Nonthaburi, Thailand, and cultivated at the Pharmacology and Toxicology Laboratory, Faculty of Veterinary Medicine, Khon Kaen University. Female mosquitoes were targeted. Mosquitoes were maintained under controlled conditions with a temperature range of 27°C–30°C and a relative humidity of 75%–80% and were fed a 10% sucrose solution until testing.

### Experimental design

The experiments were performed according to the study of Fankhauser *et al*. [14] with some modifications in the formulation administration. Briefly, the dogs were randomly divided into two groups (16 dogs per group). The control group dogs were bathed with an olive oil bath bomb without citronella essential oil, while the treatment group dogs were bathed with a bath bomb containing citronella essential oil. Before bathing, a 40 g bath bomb was dissolved in 500 mL (8% w/v) of water and then applied all over the dog (500 mL/5 kg of dog weight) and left for 5 min before wiping the fur dry. Each dog was individually caged and covered with mosquito nets post-bathing. One hundred mosquitoes were introduced into each cage. One hundred new mosquitoes were introduced after 3 and 6 h. Mosquitoes were evaluated for the presence of blood in their stomachs after 8 h by observing them under a stereomicroscope (Olympus Corporation, Japan) with a magnification of 8–50. The geometric means of the number of fed and non-fed mosquitoes were determined. The percentage of mosquito repellency was calculated using the following equation:

Repellency efficacy (%) = 100 × (m_C_ – m_T_)/m_C_

Where m_C_ is the geometric mean of fed mosquitoes in the control group and m_T_ is the geometric mean of fed mosquitoes in the treatment group.

### Skin irritation observation

Dogs were observed for signs of irritation on their bellies after a bath with a bath bomb at 0, 1, 24, 48, and 72 h, as well as every 72 h until day 15. Erythema and edema, scored from 0 to 3 based on severity, were indicators of irritation; 0 represents no lesion, 1 for mild redness, 2 for moderate redness, and 3 for severe redness or burnt skin. Edema severity on the skin was categorized from 0 to 3: 0 (no swelling), 1 (mild: clearly defined swelling), 2 (moderate: 1 mm above surrounding skin), and 3 (severe: above 1 mm, spreading beyond the area of application) [15, 16].

### Statistical analysis

Statistical analysis was performed using IBM Statistical Package for the Social Sciences v.28 Software (IBM Corp., NY, USA) with p ≤ 0.05 as significant. The Shapiro–Wilk test was used to evaluate the data’s normality. Paired samples t-test and Wilcoxon signed-rank test were used to analyze differences in citronella oil concentration, animal weight, and pH of bath bombs before and after freeze-thawing cycles, depending on the data distribution. The Mann–Whitney U test was used to analyze differences in animal weight and age, while Pearson’s Chi-square test was employed for analyzing differences in the sex ratio. Independent samples t-tests and Mann–Whitney U tests were used to analyze between-group differences in percentages of non-fed and fed mosquitoes at 3, 6, and 8 h, depending on the data distribution.

## Results

### GC-MS analysis

**Figure-1:**
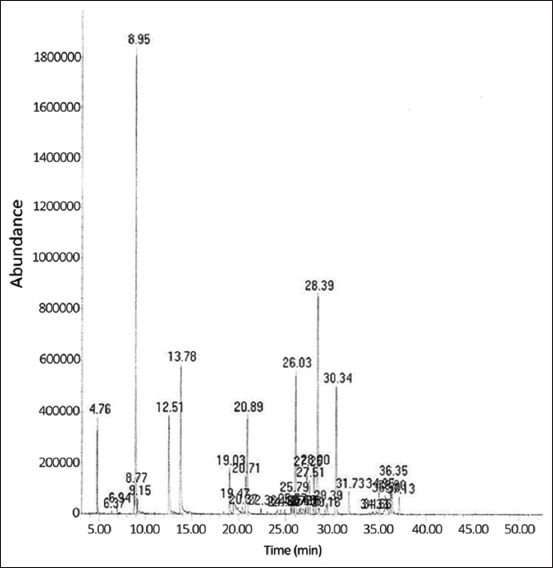
Gas chromatography-mass spectrometry spectrum of citronella essential oil.

Figure-1 depicts the GC-MS spectra of citronella essential oil. About 98.96% of the peak area was accounted by 36 compounds. The main constituent, citronellal, made up 23.38% of the peak area, with δ-cadinene contributing 12.25%, geraniol 9.09%, germacrene-D 7.90%, elemol 7.55%, citronellol 5.21%, and β-elemene 5.20%. The 12 other minor constituents (γ-cadinene, α-muurolene, *cis*-2,6-dimethyl-2,6-octadiene, isopulegyl acetate, α-cadinol, valencene, carvestrene, γ-eudesmol, isoledene, α-eudesmol, isopulegol, and D-longifolene) had individual peak areas ranging from 1.11% to 2.63% and collectively represented 21.56% of the total peak area. Seventeen of the remaining constituents displayed peaks below 1.00% and totaled 6.82% of the entire peak area (Table-1).

**Table-1:** Chemical constituent of citronella essential oil from GC-MS analysis.

No.	Chemical	Molecular formula	% of peak area	Retention time (min)
1	Citronellal	C_10_H_18_O	23.38	8.95
2	δ-Cadinene	C_15_H_24_	12.25	28.39
3	Geraniol	C_10_H_18_O	9.09	13.78
4	Germacrene-D	C_15_H_24_	7.90	26.03
5	Elemol	C_15_H_26_O	7.55	30.31
6	Citronellol	C_10_H_20_O	5.21	12.51
7	β-Elemene	C_15_H_24_	5.20	20.89
8	γ-Cadinene	C_15_H_24_	2.63	28.00
9	α-Muurolene	C_15_H_24_	2.45	27.25
10	*cis*-2,6-Dimethyl-2,6-octadiene	C_10_H_18_	2.33	19.03
11	Isopulegyl acetate	C_12_H_20_O_2_	2.17	4.76
12	α-Cadinol	C_15_H_26_O	2.05	36.35
13	Valencene	C_15_H_24_	1.92	27.51
14	Carvestrene	C_10_H_16_	1.90	20.71
15	γ-Eudesmol	C_15_H_26_O	1.34	34.95
16	Isoledene	C_15_H_24_	1.32	31.73
17	α-Eudesmol	C_15_H_26_O	1.18	36.20
18	Isopulegol	C_10_H_18_O	1.16	8.77
19	D-Longifolene	C_15_H_24_	1.11	37.13
20	α-Amorphene	C_15_H_24_	0.95	25.79
21	γ-Muurolene	C_15_H_24_	0.83	35.59
22	Neoiso (iso) pulegol	C_10_H_18_O	0.62	9.15
23	β-Cadinene	C_15_H_24_	0.61	29.39
24	Eugenol	C_10_H_12_O_2_	0.58	19.47
25	β-Bourbonene	C_10_H_24_	0.49	20.37
26	α-Selinene	C_15_H_24_	0.42	26.89
27	(+)-2-Carene	C_10_H_16_	0.32	6.94
28	α-Gurjunene	C_15_H_24_	0.30	26.45
29	Bicyclo[4.4.0]dec-1-ene, 2-isopropyl-5-methyl-9-methylene-	C_15_H_24_	0.30	26.60
30	α-Humulene	C_15_H_24_	0.26	24.46
31	(+)-Epi-bicyclosesquiphellandrene	C_15_H_24_	0.25	24.92
32	Cadina-1,4-diene	C_15_H_24_	0.25	29.16
33	α-Elemene	C_15_H_24_	0.23	25.57
34	trans-Caryophyllene	C_15_H_24_	0.16	22.36
35	Aromadendrene	C_15_H_24_	0.15	27.10
36	α-Terpinolene	C_10_H_16_	0.10	6.37

GC-MS=Gas chromatography-mass spectrometry

### Stability testing of the bath bomb formulation

**Figure-2:**
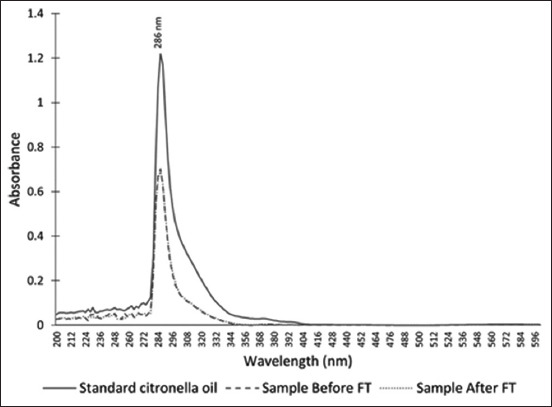
Ultraviolet-visible absorbance spectra (200–600 nm) for the standard citronella oil (10 mg/mL) and the formulation hexane extract before and after freeze-thaw testing.

**Figure-3:**
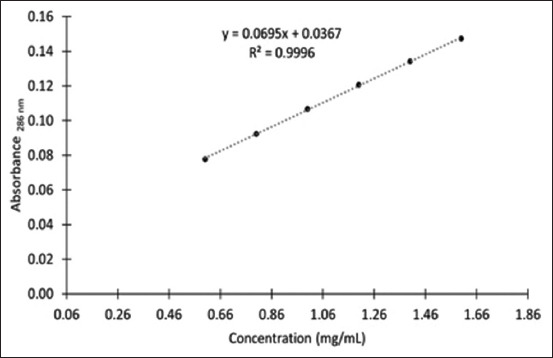
The calibration curve of citronella essential oil.

**Figure-4:**
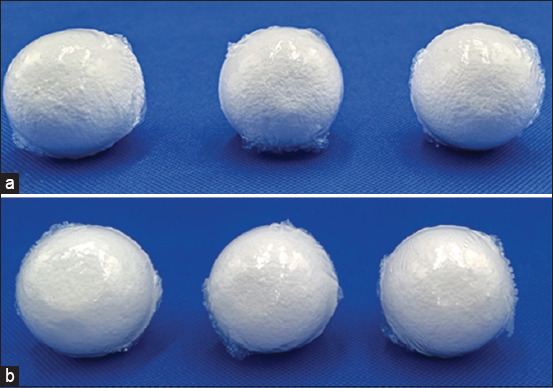
Characteristic of bath bombs (a) before and (b) after freeze-thaw test.

The standard citronella essential oil and hexane extracts of bath bomb formulations showed similar absorbance patterns (200–600 nm) with a lambda max (λ_max_) of 286 nm (Figure-2). The calibration curve of standard citronella essential oil dissolved in hexane was linear, and the equation was y = 0.0695x + 0.0367 with an r^2^ value of 0.9996 (Figure-3). Using this curve equation, the amount of citronella essential oil present in the bath bomb formulation extracts (6.08 ± 0.31% w/w) was found to correspond to the amount added during preparation. Figure-4 displays the differences in bath bomb features before and after the freeze-thaw test (FT). After six freeze-thawing cycles, the formulations maintained their original weight, pH, and color like the controls. The essential oil content in the formulations remained unchanged following freeze-thaw cycles, as shown in Table-2.

**Table-2:** The stability of the bath bomb formulation^a^.

Freeze-thaw test	Citronella oil concentration (% w/w)	Weight (g per piece)	pH (8% w/v solution at 25°C)	Color
Before	6.08 ± 0.31	38.57 ± 0.57	5.18 ± 0.03	White
After	5.90 ± 0.38	38.39 ± 0.64	5.09 ± 0.07	White
Statistic	0.515^b^	0.799^c^	0.076^c^	NA

a Values represent the mean ± SD,

b Wilcoxon signed-rank test with α=0.05,

c Paired samples t-test with α=0.05, NA=Not applicable

### Mosquito repellency effect

A total of 32 short-haired mixed-breed dogs were included in this study. The control group consisted of seven male and nine female dogs with an average age of 4.56 ± 2.19 years and an average weight of 11.00 ± 2.34 kg bathed with the bath bomb base containing olive oil instead of citronella essential oil. The treatment group consisted of nine male and seven female dogs with an average age of 5.44 ± 1.59 years and a weight of 9.00 ± 2.99 kg bathed with the citronella essential oil bath bomb (Table-3). Statistical analysis revealed no differences between the groups in weight, age, and the sex ratio of dogs (All p > 0.05).

**Table-3:** General information on experimental dogs participating in the study^a^.

Group	Age (years)	Weight (kg)	Sex
Control	4.56 ± 2.19	11.00 ± 2.34	7 male and 9 female
Treatment	5.44 ± 1.59	9.00 ± 2.99	9 male and 7 female
Statistic between groups	0.196^b^	0.086^b^	0.480^c^

a Values represent the mean ± SD,

b Mann-Whitney U test with α=0.05,

c Pearson Chi-square with α=0.05

The average percentage of blood-fed mosquitoes in the control group was similar at 3, 6, and 8 h after bathing (range 66.25%–68.75%). In the citronella essential oil treatment group, no mosquitoes were found to be blood-fed 3 h after bathing, but 20.12% were blood-fed after 6 h, and 25.31% were blood-fed 8 h after bathing. Statistical analysis indicated significant differences (p < 0.05) in the percentage of fed and non-fed mosquitoes at all time points between the experimental and control groups. The citronella bath bomb had mosquito repellency efficacy values equal to 100% at 3 h, 69.28% at 6 h, and 65.58% at 8 h (Table-4).

**Table-4:** Percentage of non-fed and fed female mosquitoes at 3, 6, and 8 h after bath bomb application.

Group	Mosquito (%)					

	3 h		6 h		8 h	
		
	Non-fed	Fed	Non-fed	Fed	Non-fed	Fed
Control (mean ± SD)	31.25 ± 4.36	68.75 ± 4.36	31.25 ± 4.81	66.25 ± 9.19	32.13 ± 5.81	67.88 ± 5.81
Control (geometric mean)	30.98	68.62	30.92	65.48	31.65	67.63
Treatment (mean ± SD)	100.00 ± 0.00	0.00 ± 0.00	78.88 ± 3.65	20.50 ± 3.97	74.56 ± 2.66	25.44 ± 2.66
Treatment (geometric mean)	100.00	0.00	78.80	20.11	74.52	25.31
Statistics between groups	<0.001^a^	<0.001^a^	<0.001^b^	<0.001^a^	<0.001^b^	<0.001^b^
Repellency efficacy (%)	100.00		69.28		65.58	

a Mann-Whitney U test with α=0.05,

b independent samples t-test with α=0.05, SD=Standard deviation

### Evaluation of the skin irritation effects

**Figure-5:**
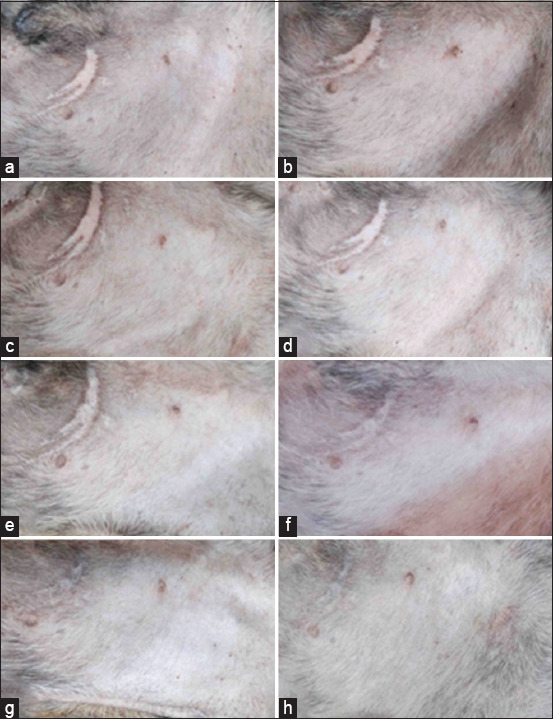
Appearance of the exposed skin area of a dog (ventral abdomen) bathed with the olive oil formulation bath bomb base. (a) Before bath bomb application, (b–h) 1 h, 1, 3, 6, 9, 12, and 15 days after application. Erythematous and edematous scores = 0 for all panels.

**Figure-6:**
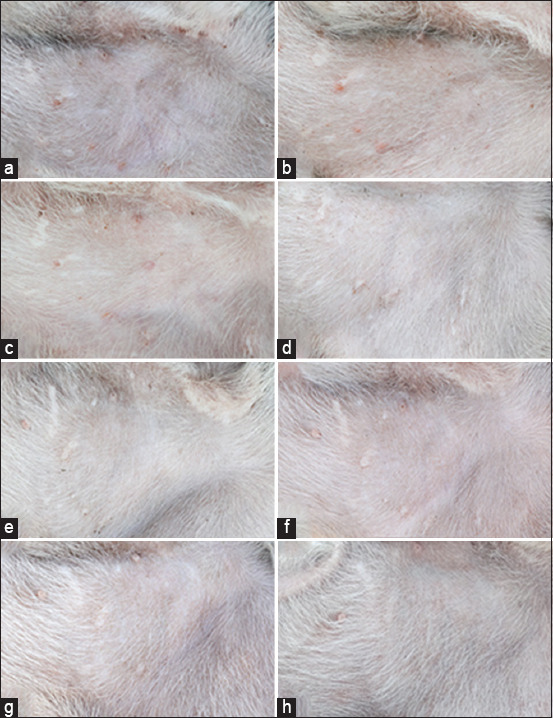
Appearance of the exposed skin area of a dog (ventral abdomen) bathed with the citronella essential oil formulation bath bomb. (a) Before bath bomb application, (b–h) 1 h, 1, 3, 6, 9, 12, and 15 days after application. Erythematous and edematous scores = 0 for all panels.

The observation of erythema and edema of the dogs in the control and treatment groups at 1 and 24 h, 3, 6, 9, 12, and 15 days after bathing revealed that no sign of irritation was found in the affected area in all tested dogs (Figures-5 and 6).

## Discussion

Essential oils typically consist of a blend of monoterpenes, sesquiterpenes, phenylpropenes, and their respective modified structures (monoterpenoids, sesquiterpenoids, and phenylpropanoids). Each substance’s identity and proportion varies, depending on the plant species, extraction method, and raw material source. GC-MS analysis revealed citronellal (23.38%), δ-cadinene (12.25%), geraniol (9.09%), germacrene-D (7.90%), elemol (7.55%), and citronellol (5.21%) as the major constituents in the citronella essential oil. This is broadly consistent with previous reports by of citronellal being the most abundant compound in citronella essential oil (range 24.57%–41.7%), followed by geraniol (15.59%–28.40%), citronellol (5.2%–11.69%), and elemol (2.25%–8.5%); however, previous studies found much less δ-cadinene (1.64%–4.34%) and germacrene D (0.80%–3.25%) [17-20].

The absorbance spectrum of an essential oil depends on the nature and proportion of its components, which absorb well in the ultraviolet wavelength range (200–400 nm). Previous studies reported the λ_max_ of citronella essential oil to be 272 nm [13] and 293 nm [21]. These values are consistent with the results of the current study, where we found a linear relationship between the absorbance and concentration of standard citronella essential oil at a λ_max_ value of 286 nm. This wavelength was employed to analyze the citronella essential oil content in bath bomb samples. FT showed that the bath bomb formulation containing citronella essential oil showed good chemical and physical stability with not <90% of the active pharmaceutical ingredient remaining after six freeze-thaw cycles compared with the initial concentration, which is considered acceptable quality [22].

The citronella oil bath bomb formulation used in the current study showed good repellent activity against mosquitoes, retaining 65.58% efficacy at 8 h with no skin irritation in the treated dogs. While there are no criteria for efficacy testing of animal mosquito repellent products, the World Health Organization uses 50% protection to determine the effective dose (ED_50_) in humans [23]. Our study’s findings on the mosquito-repelling effectiveness of citronella essential oil are consistent with results from human volunteer studies [23]. Amer and Mehlhorn [9] reported that a 20% concentration of citronella essential oil showed 75.70% repellency against *A. aegypti* for 2 h, with 52.4% repellency against *Anopheles dirus* and 100% repellency against *C. quinquefasciatus* for 8 h. A study by Tawatsin *et al*. [24] demonstrated that 25% citronella essential oil had 100% repellency against *A. aegypti* and *A. dirus* for 3 h and 100% repellency against *C. quinquefasciatus* mosquitoes for 8 h. The effectiveness of citronella essential oil in repelling mosquitoes was shown to depend on the formulation type in a report by Solomon *et al*. [10]. The study recorded repellency scores of 83.32% and 40.45% against *Anopheles arabiensis* for a 20% citronella essential oil in ethanol solution at 3 h and 6 h, respectively. The three different 15% topical formulations of citronella oil, in white soft paraffin, oil in water cream, and water-soluble wax, resulted in higher repellency scores ranging from 85.12% to 90.46% at 3 h and 45.36% to 57.15% at 6 h.

Citronella essential oil’s citronellal, citronellol, and geraniol components have been proven to deter mosquitoes. The substances identified by Eden *et al*. [25] were proven effective in repelling *A. aegypti* based on volunteer tests conducted in a laboratory setting. Citronellal, citronellol, and geraniol showed 84.00%, 86.67%, and 90.67% repellency activity at 5 min and 71.33%, 77.34%, and 78.00% repellency at 1 h, respectively. The mosquito-repellent mechanisms of essential oils are not fully understood; however, in one well-studied mechanism, the vapors of these substances interfere with the olfactory pathways of insects by acting as direct agonists for transient receptor potential A1, which affects the close-range host-seeking and blood-feeding behavior of adult female mosquitoes [26, 27].

## Conclusion

A 6% citronella-oil bath bomb effectively repelled *C. quinquefasciatus* in dogs for 100.00% at 3 h, 69.28% at 6 h, and 65.58% at 8 h. The essential oil ingredient in the developed formulation remained over 90% after undergoing six freeze-thaw cycles, demonstrating good physical and chemical stability. Further studies are needed to assess the deterrent effect on various mosquito species.

## Authors’ Contributions

SU: Conducted skin irritation test and statistical analysis. KS: Conducted GC-MS analysis. GNB: Conducted stability test of bath bomb formulation and drafted the manuscript. ET: Raised mosquitoes, tested mosquito repellent efficacy, and drafted the manuscript. JA: Developed bath bomb formulation, revised the manuscript, and contributed to mosquito repellent efficacy test. All authors have read, reviewed, and approved the final manuscript.
